# Report on the 18th International Symposium on Geo-disaster Reduction and the 4th Gu Dezhen Lecture, 20–22 November 2020, Beijing, China

**DOI:** 10.1186/s40677-021-00182-2

**Published:** 2021-05-07

**Authors:** Shengwen Qi, Ning Liang, Kongming Yan, Zili Dai, Fawu Wang

**Affiliations:** 1 Institute of Geology and Geophysics, Chinese Academy of Sciences (IGGCAS), Beijing, China; 2 College of Civil Engineering, Tongji University, Shanghai, China; 3 School of Mechanics and Engineering Science, Shanghai University, Shanghai, China

## Abstract

The 18th International Symposium on Geo-disaster Reduction (ISGdR) was held on 20–22 November in Beijing, China, focusing on the theme of “Improving the Relationship between Geoenvironment and Society”. In this symposium, a high-level Gu Dezhen Lecture and a number of keynote and invited lectures provided a platform for scientists, industrial professionals and students to share their researches and exchange novel ideas on geo-disaster reduction in a hybrid way of offline and online.

## Organizers and organizing committee

This symposium including Gu Dezhen Lecture was jointly organized by the International Consortium on Geo-disaster Reduction (ICGdR), Engineering Geology Commission, China Geology Society (ENGEO), Key Laboratory of Shale Gas and Geoengineering, Institute of Geology and Geophysics, Chinese Academy of Sciences (IGGCAS) and College of Civil Engineering, Tongji University, China.

The scientific committee was led by three co-chairs who were Sijing Wang (Academician of the Chinese Academy of Engineering (CAE)), Jiyang Wang (Academician of the Chinese Academy of Sciences (CAS)) and Fawu Wang (President of ICGdR) respectively.

The organizing committee of this Symposium included:

*Chair persons*: Qingyun Di (Vice Director of IGGCAS) and Shengwen Qi (Director of Key Laboratory of Shale Gas and Geoengineering, IGGCAS).

*Committee members*: Zili Dai, Tianming Huang, Shouding Li, Fengshan Ma, Wuwei Mao, Yanjun Shang, Kongming Yan, Jijin Yang, Hu Zheng, Zhongxia Zhou.

The secretariat of this symposium consists of Zhiqing Li, Ning Liang, Yue Zhao and Xiaolin Zhao.

## Report of the 18th ISGdR and the 4th Gu Dezhen lecture

The 18th International Symposium on Geo-disaster Reduction (ISGdR) and the 4th Gu Dezhen Lecture were focusing on the theme of “Improving the relationship between Geoenvironment and Society” and attracted more than 90 scientists and engineers offline and online from 9 countries to give 31 high-level academic presentations on the topics of geo-disasters. Besides, the livestream channel of this symposium attracted approximate 900,000 click volume. Honourably, Prof. Jianbing Peng, an Academician of CAS, was invited as the 4th Gu Dezhen Lecturer and presented a speech on research of loess. Besides, there were seven academic sessions including the Keynote Lectures and Invited Lectures. In addition, a pre-event on annual Board of Representative Meeting of ICGdR was organized as well.

## Pre-event

On 20 November, the 2020 Board of Representative Meeting of ICGdR was held at IGGCAS in Beijing. Prof. Fawu Wang, the president of ICGdR, delivered an opening address. Besides, Prof. Manchao He (Academician of CAS), Prof. Yu Liu (Director of the third Earth Science Department of National Natural Science Foundation of China (NSFC)) and Mr. Dongyao Wang (Director of the International Organization Division of the Bureau of International Cooperation of CAS) presented the meeting and expressed support for the coming symposium on behalf of various departments.

During the meeting, new membership application, financial condition and annual report of each committee (Annual Symposium Committee, Training Course Committee, Journal Editorial Committee and Award Committee) for 2020 were reported by each manager. In the end, the annual awards including 2019 Best Paper Award, Science Achievement Award, Outstanding Activity Award and Outstanding Young Scientist Award were voted by all current members.

## Plenary sessions of the symposium

### Opening ceremony

**Fig. 1:**
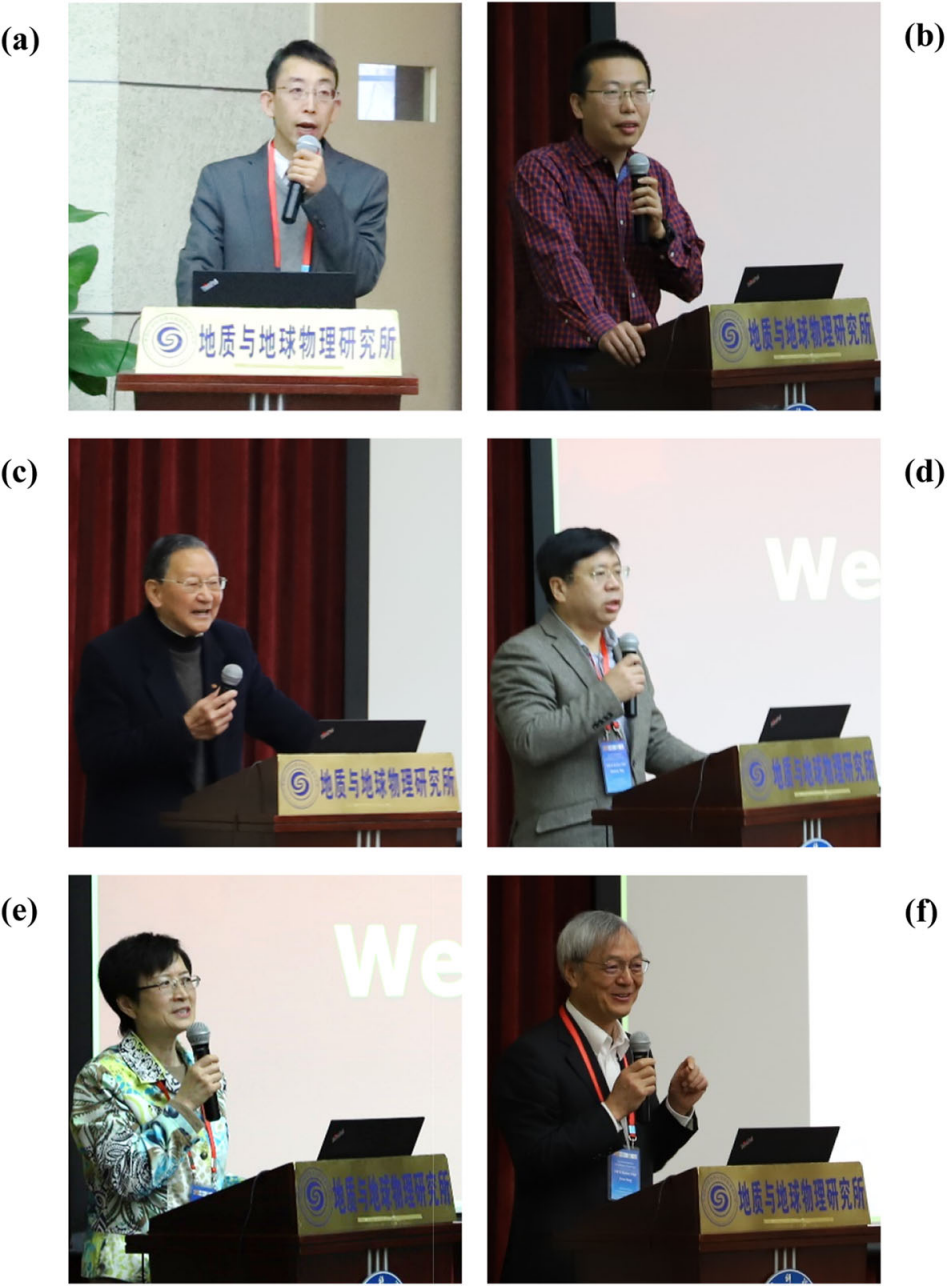
Opening speech hosted by Prof. Shengwen Qi (**a)** and presented by Dr. Xiaonan Duan (**b)**, Prof. Jiyang Wang (**c)**, Prof. Huiming Tang (**d)**, Prof. Qingyun Di (**e)** and Prof. Fawu Wang (**f)**

**Fig. 2:**
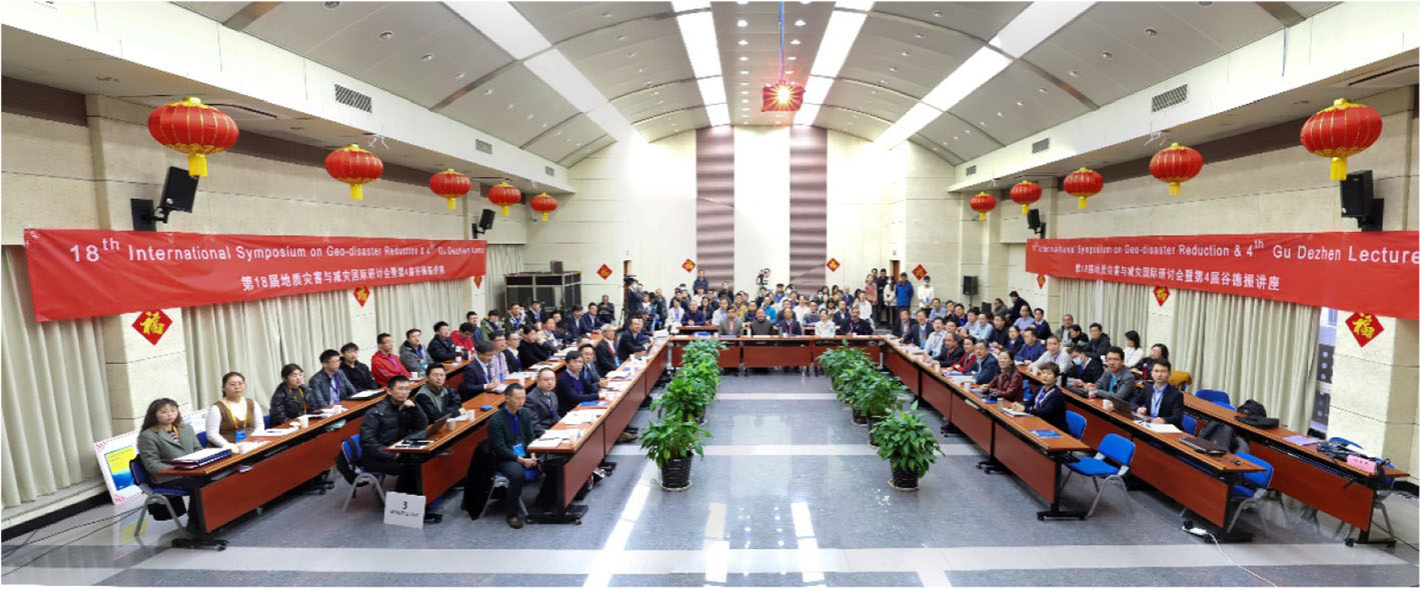
Group photo of participants of the 18th ISGdR

Prof. Shengwen Qi, Co-chair of the organizing committee, hosted the opening ceremony and declared the symposium open on behalf of the organizer, IGGCAS. In addition, five speeches were presented as the prelude (see Fig. 1). Dr. Xiaonan Duan delivered a welcome speech to all the distinguished scholars and experts from all over the world as a representative of CAS. Prof. Jiyang Wang, an academician of CAS, introduced academic stories of Prof. Gu Dezhen who was one of most well-known geologists in China. Besides, Prof. Huiming Tang (Vice Director of ENGEO) and Prof. Qingyun Di (Vice Director of IGGCAS) expressed great support and best wishes to the important events. Finally, Prof. Fawu Wang, present of ICGdR expressed his appreciation to the organizing committee and introduced the development of ICGdR, including its mission, structure, member organizations, history with exciting activity albums and so on. The opening ceremony ended up with a group photo as shown in Fig. 2.

### Certificate and award ceremony

**Fig. 3:**
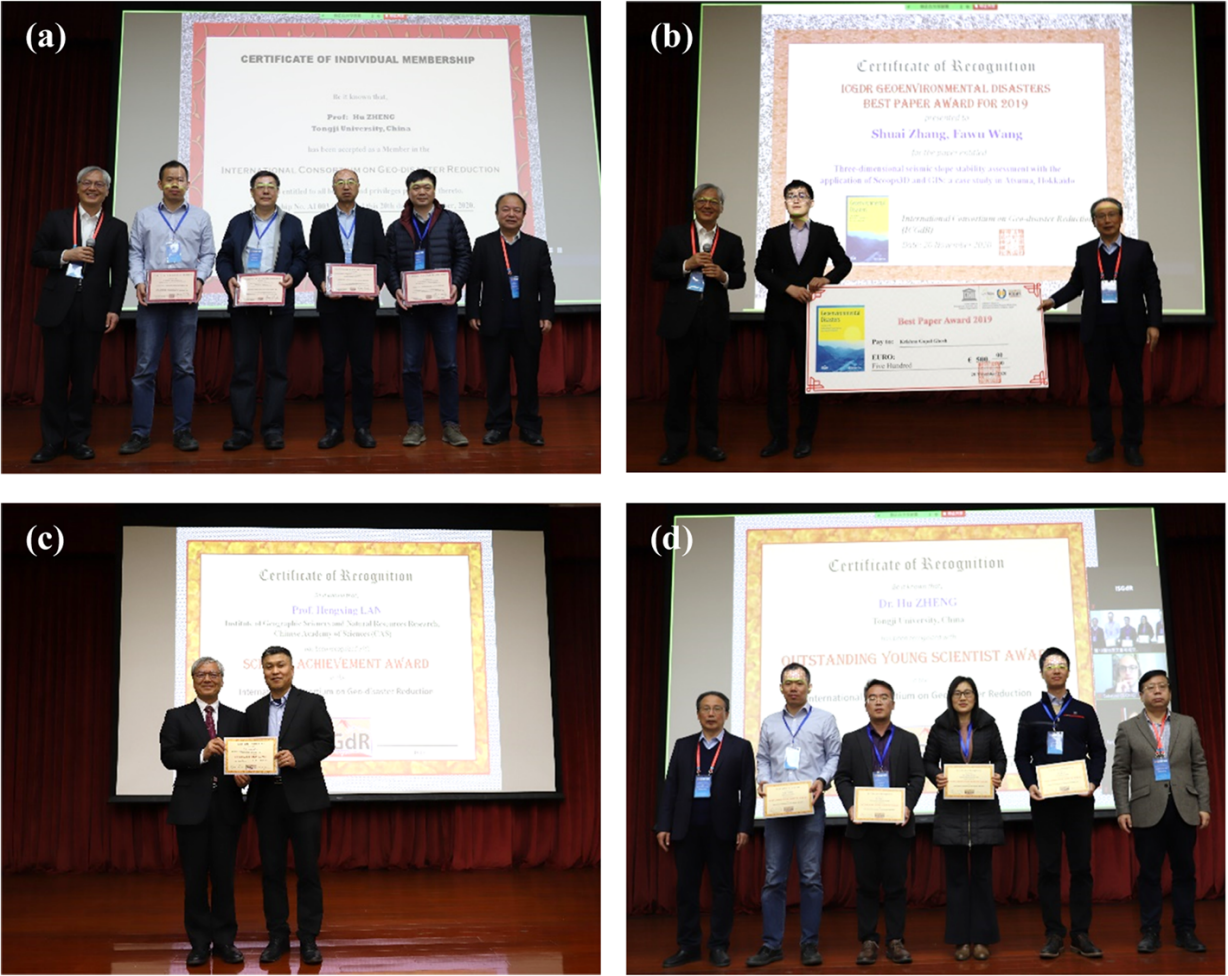
Photos of the ICGdR Awards winners (Offline only): (**a)** new ICGdR memberships, (**b)** ICGdR Geoenvironmental Disaster Best Paper Award for 2019, (**c)** Science Achievement Award and (**d)** Outstanding Young Scientist Award

Following the open ceremony, Prof. Fawu Wang hosted the certificate and award ceremony and presented certificates to part of the participants who attended the opening ceremony offline. At the beginning, Prof. Wang introduced five new ICGdR memberships and presented the certificates to them (Fig. 3a). They are Prof Shuheng Sun (Liaoning Investigation Institute of Hydrogeology and Engineering Geology), Prof. Jianhong Ye (Institute of Soil and Rock Mechanics, CAS), Prof. Wanghua Sui (IMWA China National Commission), Prof. Yonggui Chen (Tongji University) and Prof Hu Zheng (Tongji University). Besides, the 2019 Best Paper Award of ICGdR official journal Geoenvironmental Disasters was shared by two papers. They are “Spatial and temporal appraisal of drought jeopardy over the Gangetic West Bengal, eastern India” by Krishna Gopal Ghosh and “Three-dimensional seismic slope stability assessment with the application of Scoops3D and GIS: a case study in Atsuma, Hokkaido” by Shuai Zhang and Fawu Wang. Two cheques worth of 600 Euro were awarded to the authors’ representative (Fig. 3b). In addition, President Wang presented the Science Achievement Awards to Prof Sabatino Cuomo and Prof. Hengxing Lan (Fig. 3c). The Outstanding Activity Awards were presented to Isakbek Torgoev (Institute of Geomechanics and Mining, National Academy Sciences of the Kyrgyz Republic), Hans-Balder Havenith (University of Liege, Belgium) and Ranjan Kumar Dahal (Tribhuvan University, Nepal). Moreover, The ICGdR Outstanding Young Scientist Award was shared by Dr. Yufeng Wang (Southwest Jiaotong University), Dr. Yingbin Zhang (Southwest Jiaotong University), Dr. Ping Li (China Institute of Disaster Prevention), Dr. Kai Gu (Nanjing University) and Dr. Hu Zheng (Tongji University), which was recorded in Fig. 3d.

## 4th Gu Dezhen lecture

**Fig. 4:**
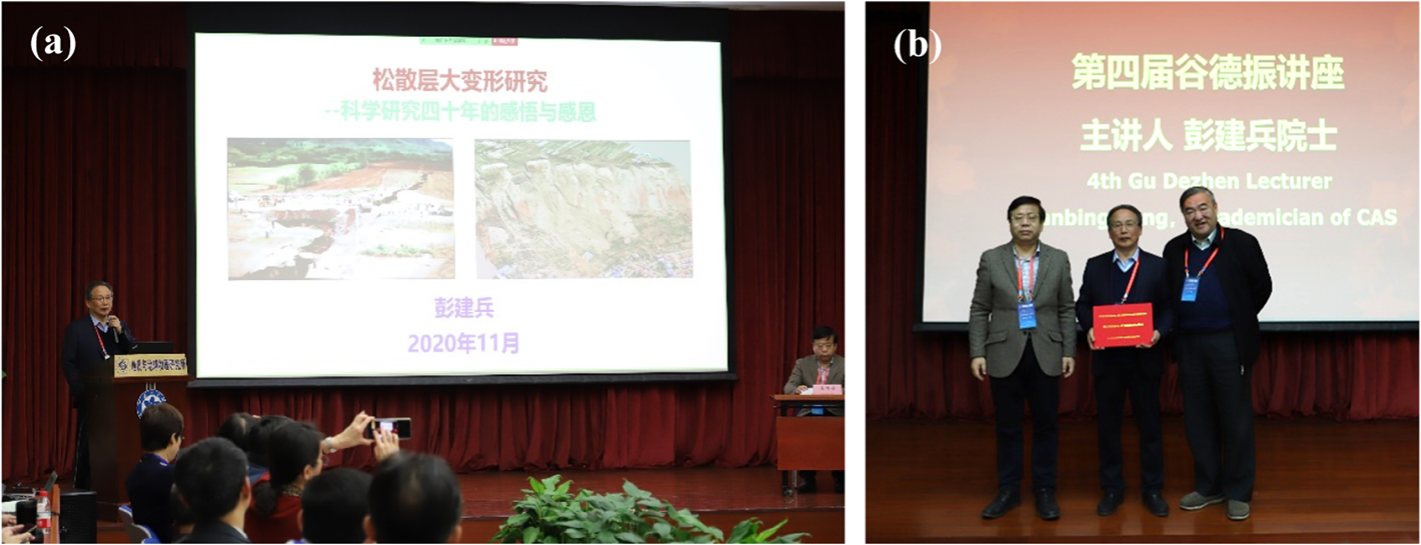
Photos of Gu Dezhen Lecturer: (**a**) Prof. Jianbing Peng and (**b**) Group photo with the hosts

Gu Dezhen Lecture, a high-level forum on geology in China, invited Prof. Jianbing Peng (Academician of CAS) as the lecturer this year. He introduced research development of the loess deformation as well as his personal academic experience, which was delivered to online audience by simultaneous interpretation. Besides, this session was hosted by Prof. Huiming Tang and Prof. Ren Wang (see Fig. 4).

## Keynote lectures

**Fig. 5:**
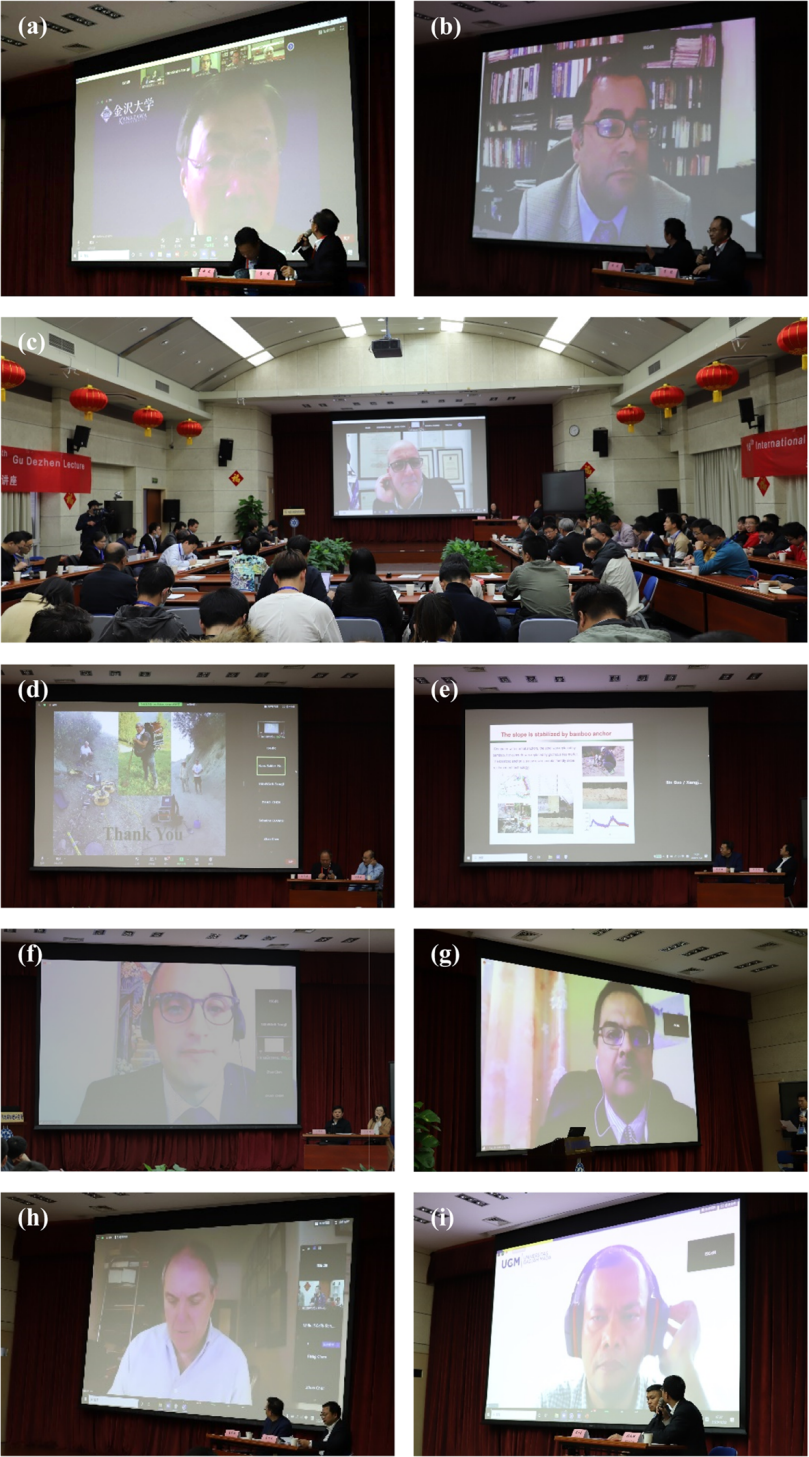
Plenary lectures presented by Prof. Masakatsu Miyajima (**a**), Prof. Binod Tiwari (**b**), Prof. Nicola Casagli (**c**), Prof. Hans-Balder Havenith (**d**), Prof. Xiangjun Pei (**e**), Prof. Sabatino Cuomo (**f**), Prof. Ranjan Kumar Dahal (**g**), Prof. Mike Winter (**h**) and Prof. Teuku Faisal Fathani (**i**) online

**Fig. 6:**
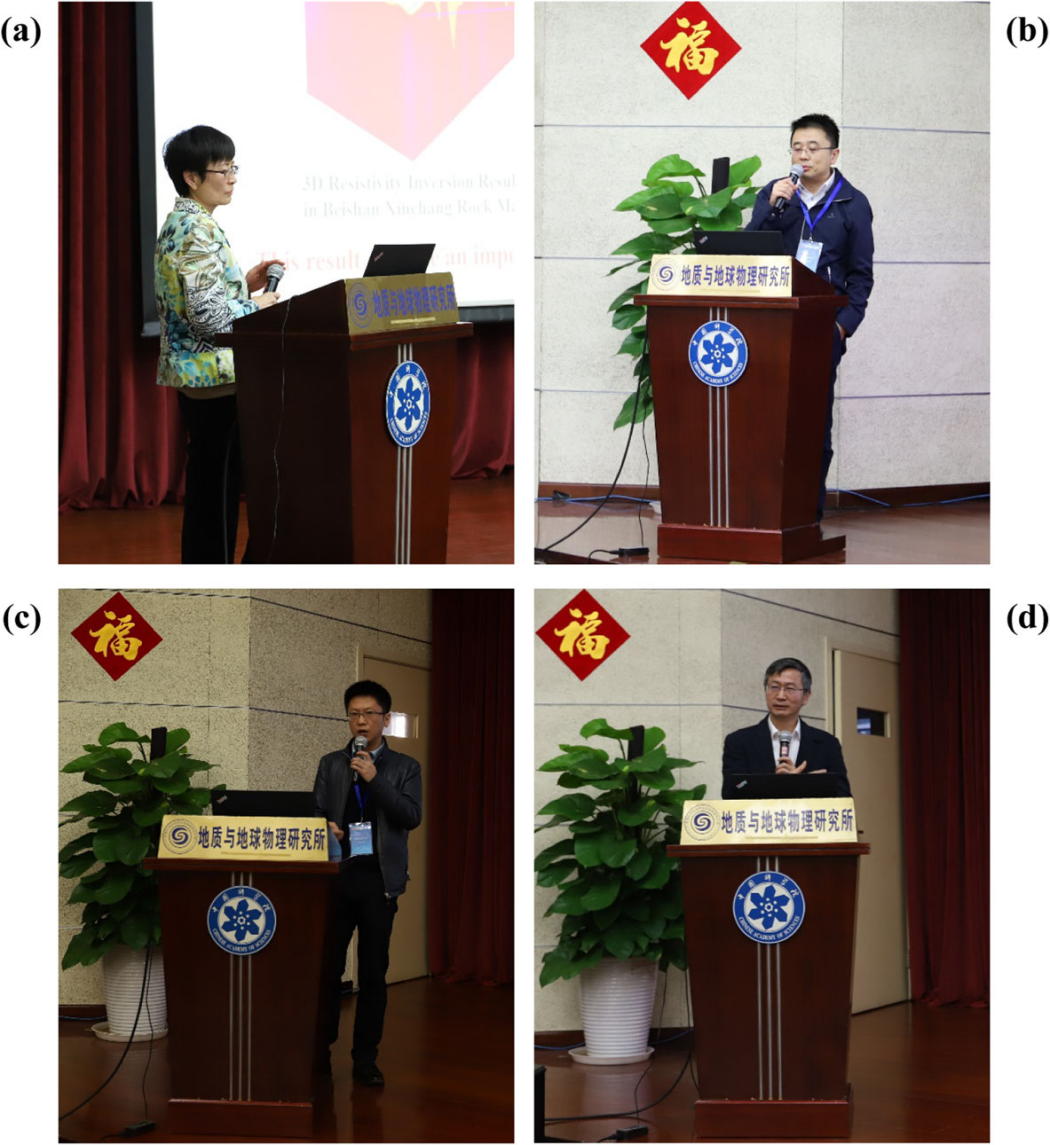
Plenary lectures presented by Prof. Qingyun Di (**a**), Prof. Yu Huang (**b**), Prof. Lijun Su (**c**) and Prof. Xiwei Xu (**d**) offline

**Fig. 7:**
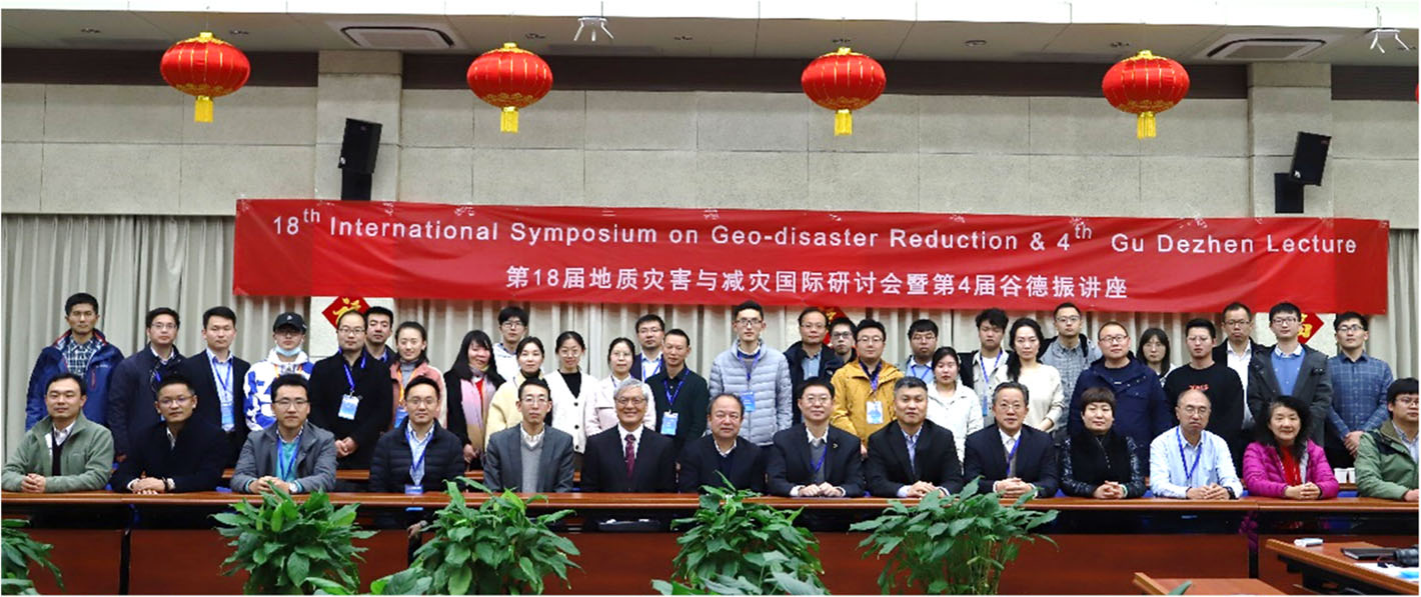
Group photo of participants at the closing ceremony

The Keynote Lectures included 13 presentations mixed with 17 invited lectures in seven sessions. The details, including topic titles, presenters (see Figs. 5, 6 and 7) and affiliations, were summarized in Table 1.

The lectures covered various topics related to the theme of this symposium, including mechanism analysis of geohazards, investigation methods, socio-economic effects and so on.

**Table 1:** Lists of the presentation in Keynote Lectures

No.	Title	Presenter	Affiliation
K-1	Large Scale Ground Flow induced by Liquefaction in the 2018 Palu Donggala Earthquake, Indonesia	Masakatsu Miyajima	Kanazawa University, Japan
K-2	Estimation of shear strength reduction in fine grained soil during earthquake loading	Binod Tiwari	California State University, USA
K-3	Radar technologies for landslide monitoring and rapid mapping	Nicola Casagli	University of Firenze, Italy
K-4	New geophysical electromagnetic exploration technology and its application in major geological engineering	Qingyun Di	IGGCAS, China
K-5	Geohazards visualised with Extended Reality tools	Hans-Balder Havenith	University of Liege, Belgium
K-6	Research on ecological restoration of seismic damage in Jiuzhaigou Valley induced by the “8.8” earthquake	Xiangjun Pei	Chengdu University of Technology, China
K-7	Multiphysics Multimaterial Modelling of Landslide-Structure-Interaction	Sabatino Cuomo	University of Salerno, Italy
K-8	Stochastic seismic analysis of slopes based on physical mechanism: Theory, experiment, and prospects	Yu Huang	Tongji University, China
K-9	Peculiarities of Deep-seated Gravitational Slope Deformations (DGSDs) in the Himalaya	Ranjan Kumar Dahal	Tribhuvan University, Nepal
K-10	Geophysical investigation on geological structures of slopes and landslide risk analysis	Lijun Su	Institute of Mountain Hazards and Environment, CAS, China
K-11	Road User, Road Infrastructure and Socio-Economic Hazards and Risks from Landslides	Mike Winter	Winter Associates, UK
K-12	Progress in active fault research and hazard mitigation action in China	Xiwei Xu	National Institute of Natural Hazards, MEMC & CAS, China
K-13	Strategic program and technological innovation for disaster risk reduction	Teuku Faisal Fathani	Universitas Gadjah Mada, Indonesia

## Closing ceremony

Followed the last keynote presentation on 22 November, the closing ceremony was hosted by Prof. Fawu Wang on behalf of ICGdR. He gave a closing speech and expressed sincere appreciation to the organizer and co-organizers for this special symposium during the worldwide COVID-19 pandemic period.

